# Respiratory Syncytial Virus induces the classical ROS-dependent NETosis through PAD-4 and necroptosis pathways activation

**DOI:** 10.1038/s41598-018-32576-y

**Published:** 2018-09-21

**Authors:** Stéfanie P. Muraro, Gabriela F. De Souza, Stephanie W. Gallo, Bruna K. Da Silva, Sílvia D. De Oliveira, Marco Aurélio R. Vinolo, Elvira M. Saraiva, Bárbara N. Porto

**Affiliations:** 1 Laboratory of Clinical and Experimental Immunology, Infant Center, School of Medicine, Pontifical Catholic University of Rio Grande do Sul (PUCRS), Porto Alegre, RS 90610-000 Brazil; 2 Laboratory of Immunology and Microbiology, School of Sciences, Pontifical Catholic University of Rio Grande do Sul (PUCRS), Porto Alegre, RS 90610-000 Brazil; 3 Laboratory of Immunoinflammation, Department of Genetics, Evolution and Bioagents, Institute of Biology, University of Campinas (UNICAMP), Campinas, SP 13083-862 Brazil; 4 Laboratory of Immunobiology of Leishmaniasis, Department of Immunology, Institute of Microbiology Paulo de Góes, Federal University of Rio de Janeiro (UFRJ), Rio de Janeiro, RJ 21941-902 Brazil

## Abstract

Respiratory syncytial virus (RSV) is a major cause of diseases of the respiratory tract in young children and babies, being mainly associated with bronchiolitis. RSV infection occurs primarily in pulmonary epithelial cells and, once infection is established, an immune response is triggered and neutrophils are recruited. In this study, we investigated the mechanisms underlying NET production induced by RSV. We show that RSV induced the classical ROS-dependent NETosis in human neutrophils and that RSV was trapped in DNA lattices coated with NE and MPO. NETosis induction by RSV was dependent on signaling by PI3K/AKT, ERK and p38 MAPK and required histone citrullination by PAD-4. In addition, RIPK1, RIPK3 and MLKL were essential to RSV-induced NETosis. MLKL was also necessary to neutrophil necrosis triggered by the virus, likely promoting membrane-disrupting pores, leading to neutrophil lysis and NET extrusion. Finally, we found that RSV infection of alveolar epithelial cells or lung fibroblasts triggers NET-DNA release by neutrophils, indicating that neutrophils can identify RSV-infected cells and respond to them by releasing NETs. The identification of the mechanisms responsible to mediate RSV-induced NETosis may prove valuable to the design of new therapeutic approaches to treat the inflammatory consequences of RSV bronchiolitis in young children.

## Introduction

Respiratory syncytial virus (RSV) is by far the most frequent cause of bronchiolitis and viral pneumonia in infants and young children worldwide^1,2^. RSV infects virtually all children by the age of 3 years, but most severe infections occur in young infants between 2 and 4 months of age^3^. Despite being highly infective, RSV does not induce an efficient immunological memory, and people are repeatedly infected throughout life. Furthermore, it has been proposed that exposure to RSV infection early in life can lead to an increased susceptibility to suffer from recurrent allergic wheezing and asthma^4^.

RSV primarily infects respiratory epithelial cells and elicits an innate immune response characterized by the release of chemokines and cytokines that promote the recruitment of immune cells from the bloodstream to the infected tissue and the activation of resident cells^5–10^. Among neutrophil chemokines, IL-8 has been shown to be released during infection, demonstrating a central role in the influx of neutrophils into the respiratory tract during RSV infection^11^.

Neutrophils constitute a first line of defense against microbes and therefore are endowed with several antimicrobial mechanisms, such as phagocytosis, degranulation, and the generation of reactive oxygen species (ROS)^12^. The most recently described mechanism is NETosis, which comprises the release to the extracellular milieu of DNA lattices, coated with granular and cytoplasmic proteins, the neutrophil extracellular traps (NETs)^13^. NET release is stimulated by a wide range of respiratory microorganisms, including viruses^14–18^.

The molecular mechanisms underlying NETosis are still poorly understood. The major route for NET release seems to be a slow lytic cell death process that occurs in time points beyond 1 hour of stimulation and is dependent on NADPH oxidase-derived ROS generation^19^. This ROS-dependent cell death program was named classical NETosis^20^. However, an early/rapid NETosis, occurring in 5–30 minutes, which is ROS-independent, was also described^21^. The specific requirements for NET release depend on the stimulus, but histone citrullination mediated by the enzyme peptidylarginine deiminase-4 (PAD-4) has been reported to be an essential step to NETosis^22,23^. On the other hand, the participation of necroptosis signaling pathways, such as receptor-interacting protein kinase 1 (RIPK1), RIPK3 and mixed lineage kinase domain-like pseudokinase (MLKL) has been controversial. GM-CSF in association with LPS or C5A has been shown to stimulate NET production independently of RIPK3 and MLKL signaling^24^, while monosodium urate crystals and particles of different sizes and shapes may activate RIPK1-RIPK3-MLKL to induce NET release^25,26^.

We have previously described that RSV virions and RSV fusion protein are both able to induce NET formation by human neutrophils^17^. Here we extend these findings by showing that RSV triggers the classical ROS-dependent NETosis. Moreover, NETs produced in response to RSV bound to virions, and expressed neutrophil elastase and myeloperoxidase in DNA threads. This mechanism is also dependent on PI3K/AKT, ERK, p38 MAPK and histone citrullination by PAD-4. Surprisingly, RSV-induced NETosis relies on RIPK1-RIPK3-MLKL activation. However, only the necroptosis executioner protein, MLKL, is necessary for LDH release induced by RSV in neutrophils. Furthermore, RSV infection of alveolar epithelial cells or lung fibroblasts stimulates the release of NETs by human neutrophils, in a virus concentration-dependent manner. Interestingly, this effect is dependent on virus replication, since the infection of epithelial cells with UV-inactivated RSV does not induce NET release. The identification of these signaling mechanisms during RSV infection-triggered classical NETosis may lead to the development of novel therapies against RSV bronchiolitis pathogenesis.

## Results

### RSV induces the classical ROS-dependent NETosis in human neutrophils

**Figure 1:**
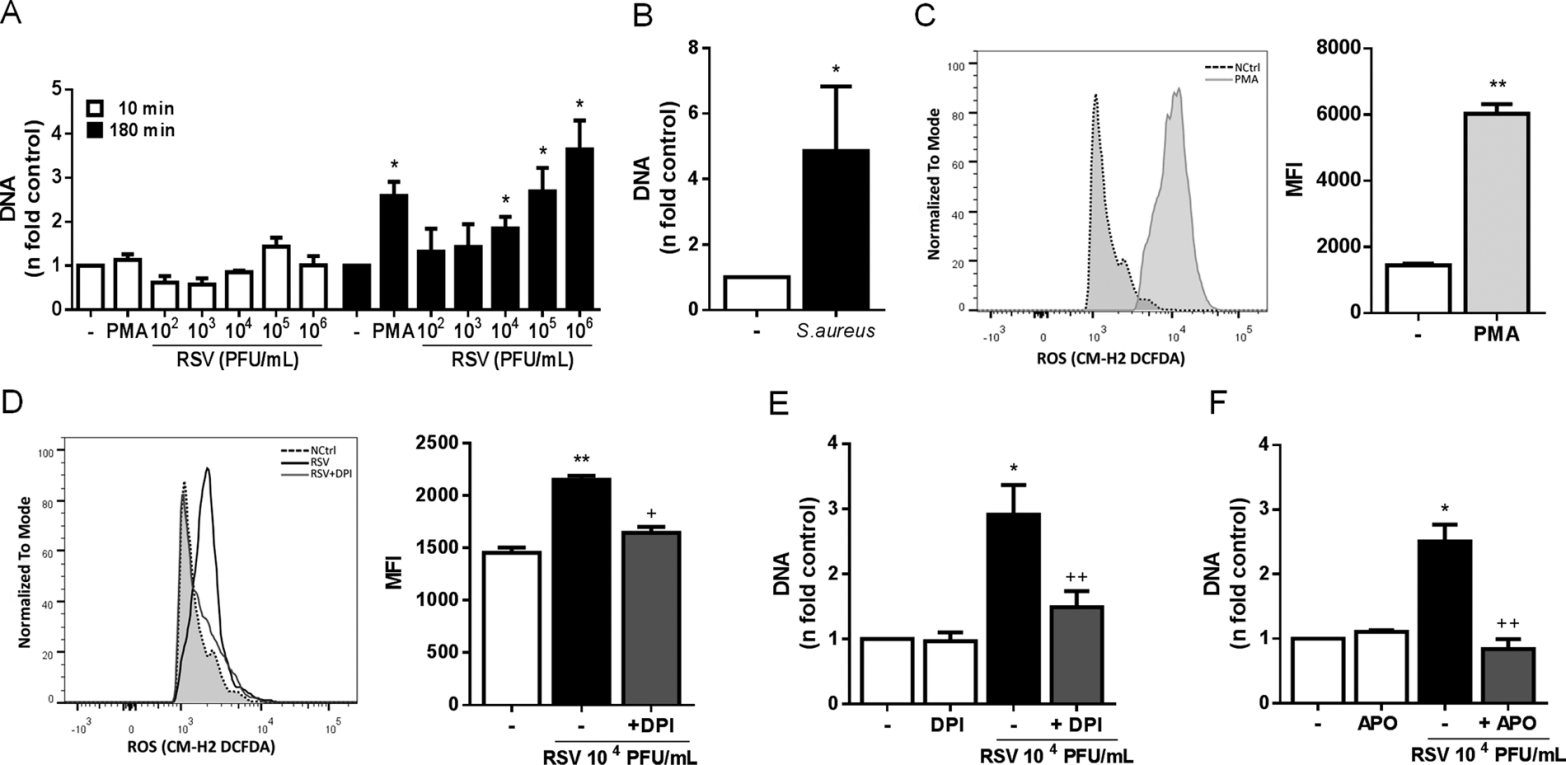
RSV induces the classical ROS-dependent NETosis in human neutrophils. (**A**) Human neutrophils (2 × 10^6^/mL) were stimulated with RSV (10^2^ – 10^6^ PFU/mL), PMA (100 nM) or left unstimulated for either 10 or 180 min. **(B)** Neutrophils (2 × 10^6^/mL) were stimulated with *Staphylococcus aureus* at bacteria to neutrophil ratios of 10:1 or left unstimulated for 10 min. NETs were quantified in culture supernatants using Quant-iT dsDNA HS kit (Invitrogen). **(C)** Neutrophils (2 × 10^6^ cells/microtube) were stimulated with PMA (50 nM) or left unstimulated for 60 min and incubated with CM-H_2_DCFDA (0.5 µM) for 30 min at 37 °C with 5% CO_2_. **(D)** Neutrophils (2 × 10^6^ cells/microtube) were pretreated with DPI (10 µM), stimulated with RSV (10^4^ PFU /mL) or left unstimulated for 60 min and incubated with CM-H_2_DCFDA (0.5 µM) for 30 min at 37 °C with 5% CO_2_. Cytosolic ROS production was measured by flow cytometry, using FACSCanto II flow cytometer. Neutrophils were pre-treated for 1 h with: **(E)** diphenyleneiodonium (DPI) or **(F)** apocynin (APO). Afterwards, cells were stimulated with RSV (10^4^ PFU/mL) for 180 min. NETs were quantified in culture supernatants using Quant-iT dsDNA HS kit. Data are representative of 3 independent experiments performed in triplicates and represent mean ± SEM. Data were analyzed with Mann Whitney test. *p < 0.05; **p < 0.01 when compared to negative control (−); ^+^p = 0.05; ^++^p < 0.05 when compared to RSV-stimulated neutrophils.

**Figure 2:**
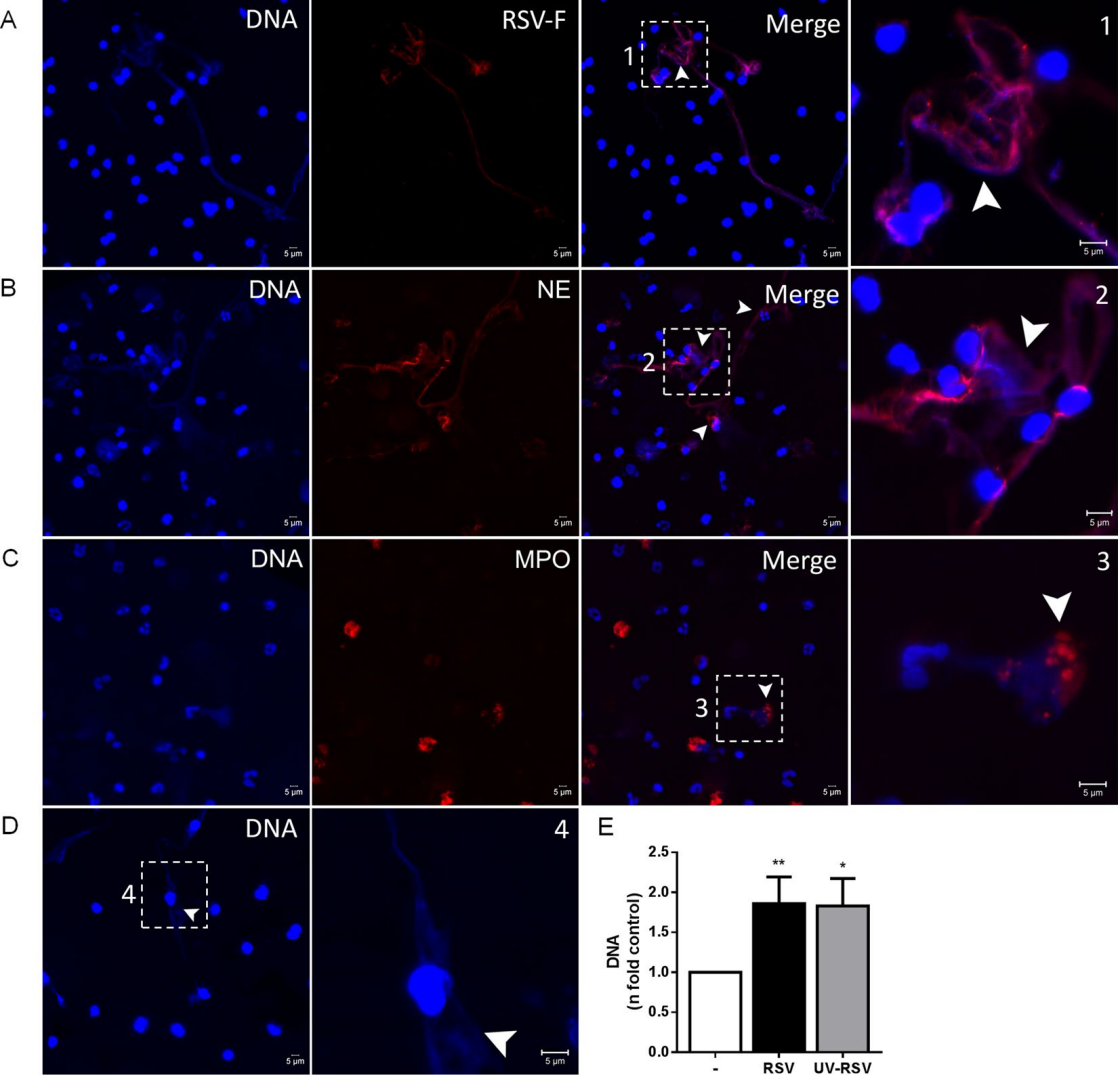
RSV triggers the release of NETs coated with NE and MPO. (**A**–**D**) Neutrophils (1 × 10^5^/300 μL) were stimulated with active RSV (10^4^ PFU/mL) or UV-inactivated RSV (10^4^ PFU/mL) for 180 min at 37 °C with 5% CO_2_ in 8-chamber culture slides. Afterwards, cells were fixed with 4% PFA and stained with **(A)** Hoechst 33342 (1:2000), anti-RSV fusion protein (1:1000) followed by anti-mouse PE antibody (1:500); **(B)** Hoechst 33342 (1:2000), anti-elastase (NE; 1:1000) followed by anti-rabbit Cy3 antibody (1:500); **(C)** Hoechst 33342 (1:2000), anti-myeloperoxidase PE (MPO, 1:1000) antibody; **(D)** Hoechst 33342 (1:2000). Overlay of the fluorescence images are shown in the penultimate panels. Arrowheads indicate the presence of extracellular DNA lattices co-localized with RSV F protein, NE and MPO, respectively. NETs were magnified four times and are numbered (1, 2, 3 and 4) on the right side. Images are representative of 3 independent experiments. Images were taken in a Zeiss LSM 5 Exciter microscope. Scale bars = 5 μm. **(E)** Neutrophils (1 × 10^6^/mL) were stimulated with active RSV (10^4^ PFU/mL) or UV-inactivated RSV (10^4^ PFU/mL) for 180 min at 37 °C with 5% CO_2_. Afterwards, NETs were quantified in culture supernatants using Quant-iT dsDNA HS kit (Invitrogen). Data are representative of 2 independent experiments performed in triplicates and represent mean ± SEM. Data were analyzed with Mann Whitney test. *p < 0.05, **p < 0.01 when compared to non-infected control (−).

We and others have previously demonstrated that RSV is able to stimulate the release of NETs from human and bovine neutrophils, respectively^16,17^. However, the exact mechanisms underlying this phenomenon are not fully characterized. We sought to elucidate whether RSV would induce the early/rapid and/or classical NETosis, by stimulating human neutrophils for either 10 or 180 minutes. RSV was only able to stimulate NETs release after 180 minutes, in a concentration-dependent manner **(**Fig. 1A**)**. As a control, we stimulated neutrophils with PMA for the same time points. PMA also induced the classical NETosis, after 180 minutes of incubation **(**Fig. 1A**)**. Additionally, in a control experiment for rapid NETs release, neutrophils incubated with *Staphylococcus aureus* for 10 minutes were able to produce NETs (Fig. 1B). Since ROS generation by NADPH oxidase complex has been shown to be an essential process during the classical mechanism of NETosis^19,27,28^, we investigated whether RSV would stimulate ROS production by human neutrophils. First, we used PMA since it is a well-known inducer of ROS from neutrophils and it potently triggered ROS generation (Fig. 1C). In addition, RSV induced ROS generation by neutrophils and the pretreatment of cells with NADPH oxidase inhibitor, DPI, abrogated this effect (Fig. 1D), indicating that RSV stimulates ROS generation through NADPH oxidase activation. To evaluate the involvement of ROS on RSV-induced classical NETosis, human neutrophils were pretreated with two different oxidase inhibitors, apocynin (APO) and DPI. Pretreating neutrophils with DPI profoundly inhibited NET release elicited by RSV **(**Fig. 1E**)**. Similarly, treatment with APO abolished RSV-stimulated classical NETosis **(**Fig. 1F**)**. Next, we performed confocal laser scanning microscopy to detect whether RSV was bound to the NETs. Consistently, our results revealed that NETs produced in response to RSV bound to the virions, as visualized by the co-localization of RSV F protein with extracellular DNA **(**Fig. 2A**)**. Additionally, RSV induced the release of NETs coated with the granular proteins NE **(**Fig. 2B**)** and MPO **(**Fig. 2C**)**, as visualized by immunostaining. Further, we evaluated the effect of UV-inactivated RSV on NETs release, and found that UV-RSV was still able to trigger NETs release, as observed by confocal microscopy (Fig. 2D) and extracellular DNA quantification (Fig. 2E). Together, these results indicate that RSV induces the classical ROS-dependent NET release, with DNA lattices expressing NE and MPO.

### RSV promotes NET release dependently of PI3K/AKT, ERK and p38 MAPK activation

**Figure 3:**
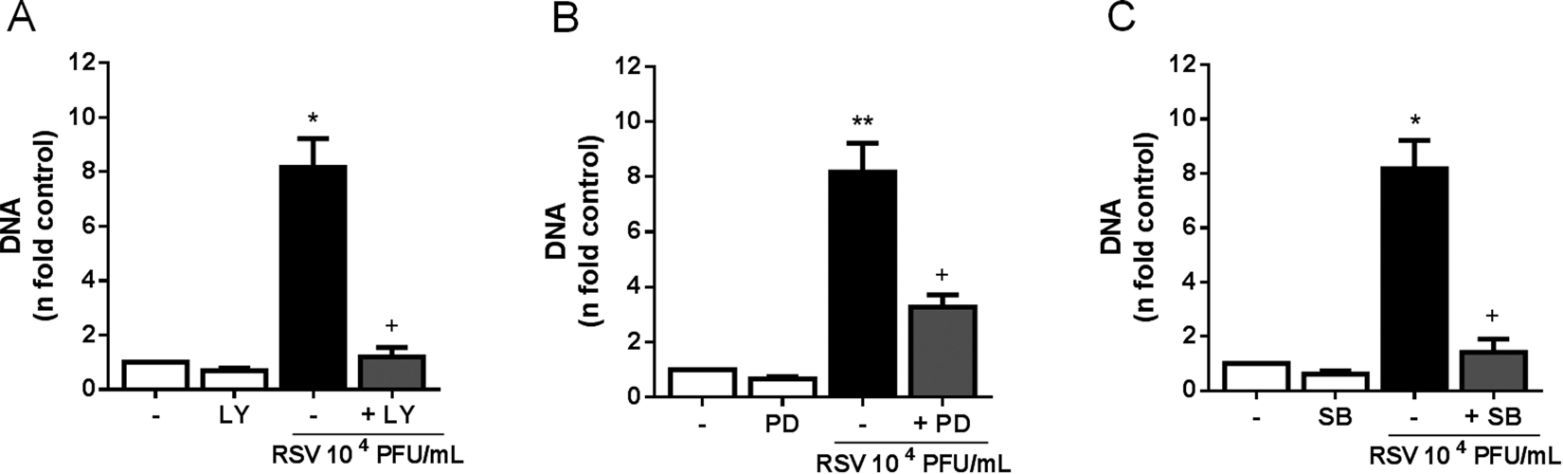
Treatment with PI3K/AKT, ERK and p38 MAPK inhibitors significantly reduce RSV-triggered NETosis. Human neutrophils (2 × 10^6^/mL) were pre-treated for 1 h with: (**A**) LY294002 (50 µM), **(B)** PD98059 (30 µM) or **(C)** SB203580 (10 µM) and stimulated with RSV (10^4^ PFU/mL) for 180 min. Afterwards, NETs were quantified in culture supernatants, using Quant-iT dsDNA HS kit. Data are representative of at least 3 independent experiments performed in triplicates and represent mean ± SEM. Data were analyzed with unpaired Student’s t-test or Mann Whitney test. *p < 0.05, **p < 0.01 when compared to non-infected control (−); ^+^p < 0.05 when compared to RSV-stimulated neutrophils.

Previous studies have shown that AKT, ERK and p38 MAPK are important to direct neutrophils to NETosis^29–31^. Thus, we decided to investigate the role of these proteins on NET production elicited by RSV, by treating neutrophils with selective inhibitors of PI3K/AKT, ERK and p38 MAPK. Pretreating the cells with PI3K/AKT inhibitor, LY294002, abrogated RSV-induced NET formation **(**Fig. 3A**)**. Treatment with PD98059, ERK inhibitor, as well as with SB203580, p38 MAPK inhibitor, significantly impaired NET release stimulated by the virus in human neutrophils **(**Figs. 3B,C**)**. These data suggest that NETosis induced by RSV occurs through a mechanism dependent on specific kinases activation.

### Essential role for PAD-4 on classical NETosis induced by RSV infection

**Figure 4:**
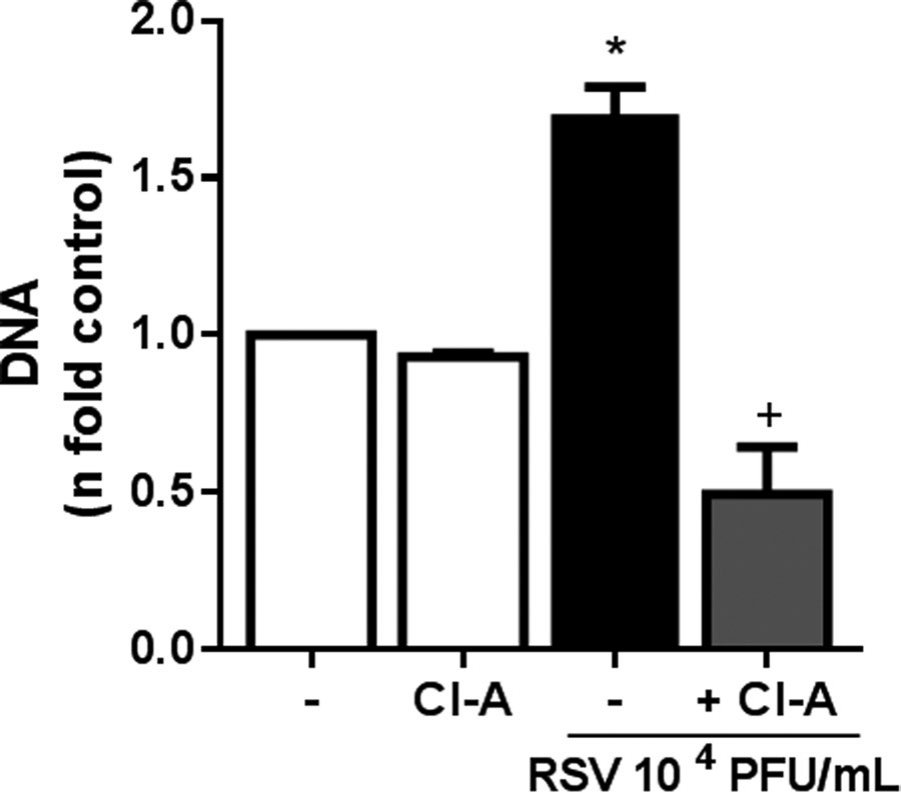
Chloroamidine abolished the classical NETosis induced by RSV. Human neutrophils (2 × 10^6^/mL) were pre-treated for 1 h with Cl-amidine (Cl-A, 12 µM) and then stimulated with RSV (10^4^ PFU/mL) for 180 min. Afterwards, NETs were quantified in culture supernatants, using Quant-iT dsDNA HS kit (Invitrogen). Data are representative of 2 independent experiments performed in triplicates and represent mean ± SEM. Data were analyzed with Mann Whitney test. *p < 0.01 when compared to non-infected control (−); ^+^p < 0.01 when compared to RSV-infected neutrophils.

Histone citrullination by the enzyme peptidylarginine deiminase-4 (PAD-4) is necessary to chromatin decondensation and has been shown to be a central step to NET formation^23^. Therefore, we sought to characterize the role of this enzyme during RSV-induced classical NETosis. Indeed, neutrophils treated with chloroamidine (PAD-4 inhibitor) failed to release NETs in response to RSV infection **(**Fig. 4**)**, indicating that PAD-4 plays an essential role on NET release promoted by RSV.

### RIPK1, RIPK3 and MLKL are involved on RSV-driven NETosis

**Figure 5:**
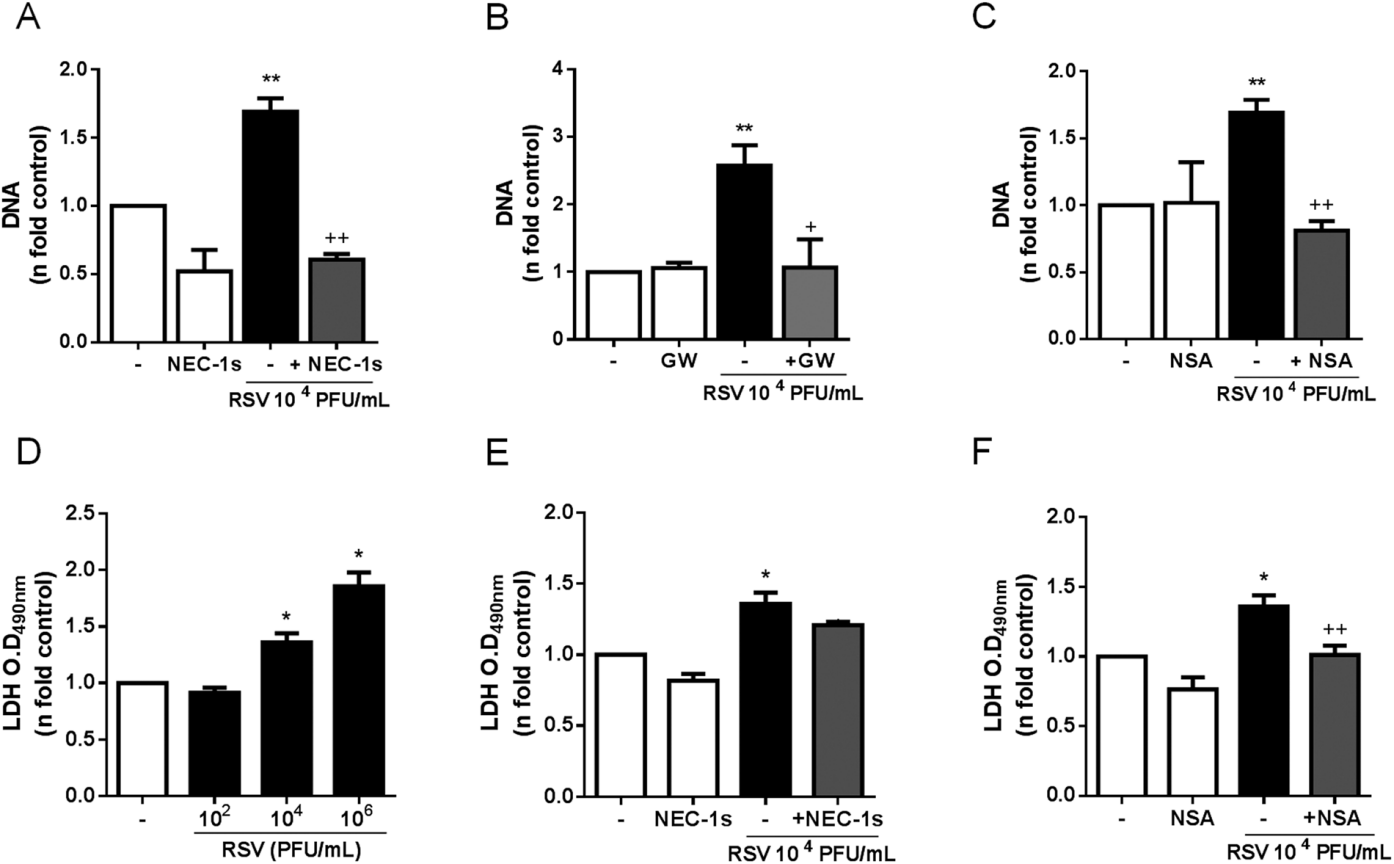
RIPK1, RIPK3 and MLKL are involved on RSV-driven NETosis. Neutrophils were pretreated with: **(A)** necrostatin-1s (Nec-1s; 50 µM), **(B)** GW42X (GW; 0.5 µM) or **(C)** necrosulfonamide (NSA; 5 µM) for 1 h and stimulated with RSV (10^4^ PFU/mL) for 180 min. After this period, NETs were quantified in culture supernatant, using Quant-iT dsDNA HS kit. **(D)** Supernatants from neutrophils stimulated with RSV (10^2^–10^6^ PFU/mL) for 180 minutes were collected and assayed for LDH release. **(E,F)** Supernatants from neutrophils pretreated for 1 h with Nec-1s (50 µM) or NSA (5 µM) and stimulated with RSV (10^4^ PFU/mL) for 180 minutes were collected and assayed for LDH release. Cytotoxicity was measured at 490 nm. Data are representative of at least 2 independent experiments performed in triplicates and represent mean ± SEM. Data were analyzed with unpaired Student’s t-test or Mann Whitney test. *p < 0.05, **p < 0.01 when compared to non-infected control (−); ^+^p < 0.05, ^++^p < 0.01 when compared to RSV-stimulated neutrophils.

During classical NETosis, neutrophils are thought to enter a cell death process that culminates with the release of NETs, as the cell membrane breaks^19^. However, the participation of necroptosis signaling pathways during NETosis has been a matter of debate. GM-CSF in association with LPS or C5A has been shown to stimulate NET production independently of RIPK3 and MLKL signaling^24^, while monosodium urate crystals and particles of different sizes and shapes may activate RIPK1-RIPK3-MLKL to induce NET release^25,26^. To investigate the importance of necroptosis signaling pathways during RSV infection-induced NETosis, we incubated neutrophils with selective inhibitors of RIPK1, RIPK3 and MLKL proteins and infected them with RSV. Inhibiting RIPK1 kinase activity with NEC-1s completely suppressed NET production stimulated by RSV infection in human neutrophils **(**Fig. 5A**)**. Likewise, suppression of RIPK3 by GW42X and necrosulfonamide (NSA) inhibition of MLKL blocked RSV-induced NETosis **(**Figs. 5B,C**)**.

Next, we were interested in further elucidate the effect of RSV infection on neutrophil necrosis. We measured lactate dehydrogenase (LDH) activity as a marker of plasma membrane rupture in supernatants of neutrophils exposed to RSV. We stimulated neutrophils with increasing concentrations of RSV and measured LDH in supernatants. RSV-neutrophil interaction was able to induce the release of LDH in a concentration-dependent manner **(**Fig. 5D**)**. Surprisingly, pretreating neutrophils with NEC-1s did not affect LDH release induced by RSV **(**Fig. 5E**)**. On the other hand, the pretreatment of cells with NSA significantly reduced LDH release triggered by the virus **(**Fig. 5F**)**. Taken together, these data suggest that RIPK1 kinase activity mediates RSV-induced NETosis, but it is not necessary for LDH release during RSV infection. The executioner protein of necroptosis, MLKL, is essential for both RSV-induced NETosis and LDH release.

### RSV infection of alveolar epithelial cells and lung fibroblasts promotes NET release by human neutrophils

**Figure 6:**
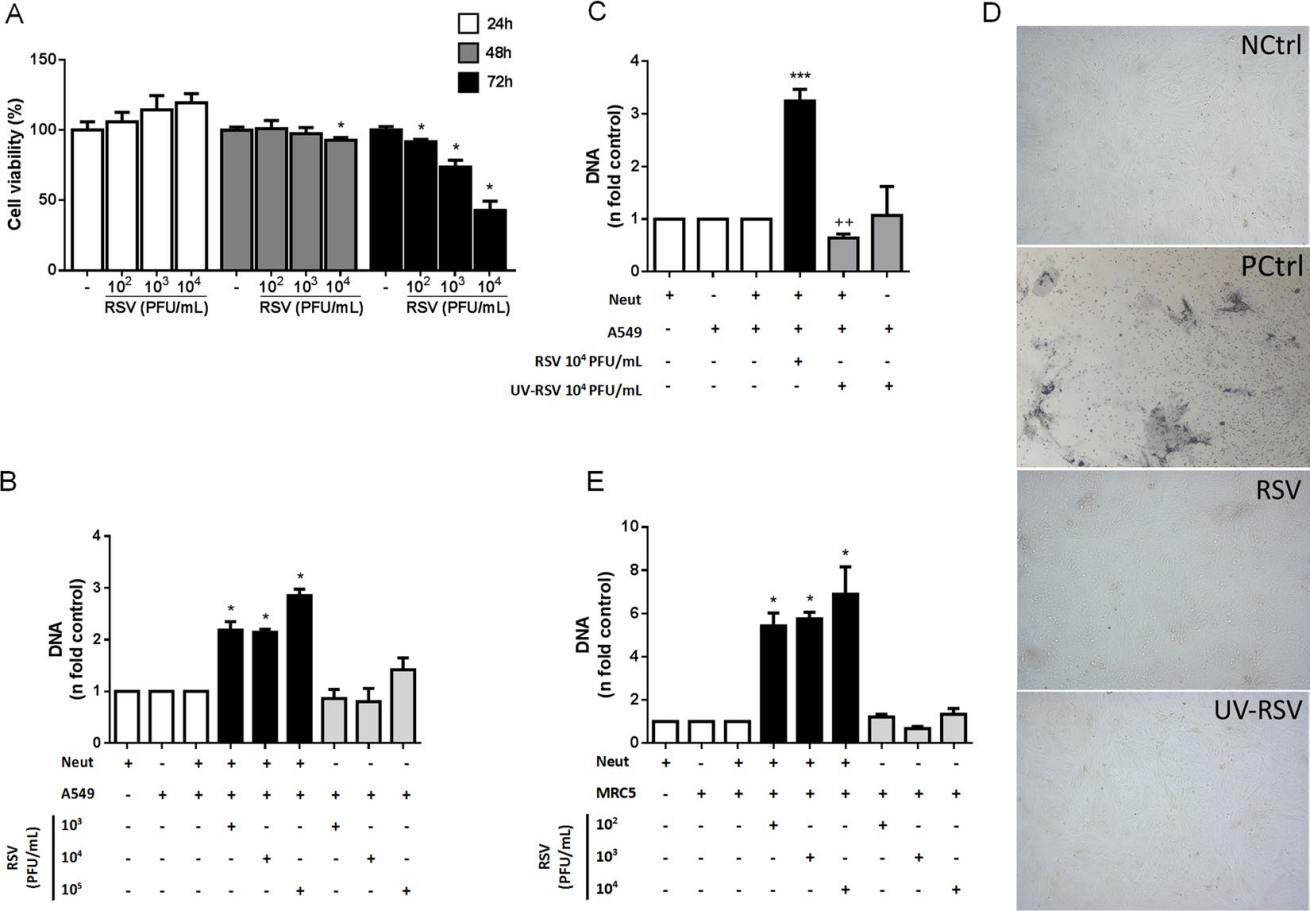
RSV infection of alveolar epithelial cells and lung fibroblasts promotes NET release by human neutrophils. (**A**) A549 cells (1 × 10^5^/mL) were infected with RSV (10^2^–10^4^ PFU/mL) for 24, 48 and 72 hours at 37 °C under 5% CO_2_ for cell viability assay with MTT. Cell viability was measured at 490 nm. **(B)** A549 cells (1 × 10^5^/mL) were infected with RSV (10^2^–10^4^ PFU/mL) for 2 h at 37 °C with 5% CO_2_. Afterwards, medium was replaced and infected cells were incubated for 48 h at 37 °C with 5% CO_2_. After this period, the medium was replaced again and neutrophils (5 neutrophils: 1 epithelial cell ratio) were added to the culture. The co-culture was maintained for 150 min at 37 °C under 5% CO_2_. **(C)** A549 cells (1 × 10^5^/mL) were infected with active RSV (10^4^ PFU/mL) or UV-inactivated RSV (10^4^ PFU/mL) for 2 h at 37 °C with 5% CO_2_. Afterwards, medium was replaced and infected cells were incubated for 48 h at 37 °C with 5% CO_2_. After this period, the medium was replaced again and neutrophils (5 neutrophils: 1 epithelial cell ratio) were added to the culture. The co-culture was maintained for 180 min at 37 °C under 5% CO_2_. **(D)** The supernatants of RSV-infected, uninfected or UV-RSV-treated A549 cells were assayed to assess viral titer, as described in the Methods section. Lysis plate titration was performed using an anti-RSV antibody. Images were magnified ten times. Images are representative of 2 independent experiments. **(E)** MRC5 cells (5 × 10^4^/mL) were infected with RSV (10^2^–10^4^ PFU/mL) for 2 h at 37 °C with 5% CO_2_. Afterwards, medium was replaced and infected cells were incubated for 48 h at 37 °C with 5% CO_2_. After this period, the medium was replaced again and neutrophils (5 neutrophils: 1 fibroblast ratio) were added to the culture. The co-culture was maintained for 150 min at 37 °C under 5% CO_2_. NETs were quantified in culture supernatants, using Quant-iT dsDNA HS kit. Data are representative of at least 3 independent experiments performed in triplicates and represent mean ± SEM. Data were analyzed with unpaired Student’s t-test or Mann Whitney test. *p < 0.05 when compared to each respective non-infected cell control (**A**) *p < 0.05, ***p < 0.001 when compared to non-infected A549/MRC5 + Neut (**B**,**C**,**E**) ^++^p < 0.001 when compared to RSV-infected A549 + Neut (**C**). Neut = Neutrophils.

Since alveolar epithelial cells constitute the primary target for RSV infection^32,33^, we decided to verify whether neutrophils would release NETs in response to RSV infection of A549 cell line. First, we evaluated the cytotoxic effect of virus on A549 cells by infecting them with increasing concentrations of RSV for different time points. A549 cells viability remained high (around 100%) until 48 h of infection at all virus concentrations tested (10^2^, 10^3^ and 10^4^ PFU/mL). From 72 h of infection, at 10^3^ and 10^4^ PFU/mL of RSV, cell viability significantly decreased to 75% and less than 50%, respectively **(**Fig. 6A**)**. Then, to ensure that 100% of alveolar epithelial cells would be alive during infection, in the subsequent experiments we exposed the cells to RSV for 48 h. To analyze the effect of RSV infection of A549 cells on NET release by human neutrophils, A549 cells were infected for 48 h, medium was replaced to remove free virus particles and neutrophils were only exposed to infected epithelial cells. Neutrophils were able to produce NETs in response to RSV infection of alveolar epithelial cells **(**Fig. 6B**)**. Interestingly, this effect was abolished when cells were stimulated with UV-inactivated RSV (UV-RSV) **(**Fig. 6C**)**, indicating that an active RSV infection of epithelial cells is necessary to induce NETosis. To ensure that neutrophils were reacting only to infected epithelial cells and not to recently released virus particles, at the end of incubation we assessed viral titer in the supernatants of A549 cells by a plaque assay (as described in the Methods section). In fact, the supernatants of RSV-infected alveolar epithelial cells did not show syncytia formation, as well as the supernatants of UV-RSV-stimulated alveolar epithelial cells (Fig. 6 D). On the other hand, the supernatants of RSV-infected alveolar epithelial cells that were not replaced presented many syncytia (PCtrl) (Fig. 6 D). To rule out a possible effect of tumor cells on NET induction, we used MRC-5 cell line, a normal lung fibroblast line. RSV infection of lung fibroblasts was capable of inducing NETosis, in a virus concentration-dependent fashion **(**Fig. 6E**)**. Importantly, non-infected A549 or MRC-5 cells did not induce NET release from neutrophils (Figs. 6B and 6E). Altogether, these data suggest that neutrophils are able to recognize alveolar epithelial cells and lung fibroblasts actively infected with RSV and to respond by releasing NETs.

## Discussion

We describe here that increasing concentrations of RSV trigger NET release from human neutrophils after 180 minutes of stimulation. Moreover, RSV stimulates ROS generation through the activation of NAPH oxidase complex. With the use of two different NADPH oxidase inhibitors, apocynin and diphenyleneiodonium, we show that RSV-induced NET release is dependent on NADPH oxidase-derived ROS generation. To date, two main mechanisms of NET formation have been proposed in the literature. The most studied route for NET production appears to be a slow lytic cell death mode, taking place in 180–240 minutes of stimulation, on which ROS produced by a functional NADPH oxidase are required^19^. In this process, neutrophils enter an irreversible cell death program that initiates with the loss of the characteristic lobular shape of the nucleus. At later time points, the nuclear envelope and the membranes of the granules dismantle, allowing the mixture of nuclear, citosolic and granular components. After the chromatin and cell components are mixed, the plasma membrane breaks and NETs are extruded^19^. As the majority of NET-inducing stimuli relies on ROS production, this beneficial cell death mode was coined classical “NETosis”^20^. However, in a NADPH oxidase-independent manner, neutrophils have been shown to extrude NETs after as little as 5–30 minutes in response to *Staphylococcus aureus*^21^, *Candida albicans*^34^ and *Leishmania amazonensis*^35^. Differently, this was not the case for RSV, which did not stimulate the early/rapid NETosis. One plausible explanation for such lack of effect is the time required for RSV infection of target cells, which starts in 2–3 hours after exposure^36^.

It has been previously shown that NETs have the ability to capture viral particles, as is the case for HIV-1^37^. Moreover, NETs have also been reported to attach to RSV particles^16^. However, in both of these studies, viruses were exposed to PMA-induced NETs. Using a more physiological approach, we stimulated neutrophils directly with RSV to visualize virions bound to the NETs. We demonstrate that upon interaction with RSV, human neutrophils release NETs and that RSV virions were trapped by NET threads, as shown by the co-localization of RSV F protein with extracellular DNA. In addition, RSV triggered the formation of NETs decorated with proteins from the azurophilic granules, NE and MPO. These proteins have been shown to modulate NET release^38,39^ and to exert potent antimicrobial activities. MPO expressed on PMA-induced NETs was able to inactivate HIV-1^37^. Nonetheless, whether MPO and NE present in RSV-induced NETs are able to inactivate the virus is yet to be elucidated. Interestingly, UV-inactivated RSV was still able to induce NETs release. This effect is likely to be due to RSV F protein interaction with TLR-4, as we have previously shown^17^.

It has been reported that AKT is essential to direct PMA-induced NADPH oxidase-dependent NETosis^31^. To address the importance of PI3K/AKT cascade on RSV-triggered NETosis, we incubated neutrophils with a PI3K inhibitor that leads to the dephosphorylation of AKT^40^. Our results point out that the axis PI3K/AKT has a fundamental role on NETosis stimulated by RSV, since LY294002 abolished NET release induced by the virus. Similarly, recent evidence from the literature shows that different stimuli, such as monosodium urate crystals and viable opsonized *S. aureus*, promoted NETosis via PI3K activation^41^. Interestingly, upon the interaction with neutrophils, *Leishmania amazonensis* activated mainly the isoforms PI3Kγ and PI3Kδ to trigger NET production^42^. Besides PI3K/AKT, signaling activation by ERK and p38 MAPK downstream of NADPH oxidase has been shown to be necessary to NETosis^29,30,43^ and we have previously reported that RSV F protein induces the phosphorylation of ERK and p38 to stimulate NET formation^17^. In this sense, we demonstrate here that RSV-incited classical NETosis is mediated by the activation of ERK and p38 MAPK. More recently, AKT, ERK and p38 were shown to be major kinases responsible to regulate the transcription of genes involved in chromatin decondensation, necessary to PMA-driven NADPH oxidase-dependent NETosis^44^. Thus, RSV might activate PI3K/AKT, ERK and p38 to control the transcription of specific genes during NETosis.

PAD-4-mediated conversion of arginine to citrulline in histones, also known as histone citrullination, is crucial to chromatin decondensation during NETosis^22,23,45^. In fact, pharmacological inhibition of PAD-4 profoundly decreased chromatin decondensation and NET release in response to ionomycin, *Shigella flexneri* and *L. amazonensis*^22,35^. Likewise, PAD-4-deficient neutrophils were shown to be unable to citrullinate histones, decondense chromatin and consequently, release NETs^14,23^. Therefore, we investigated the participation of PAD-4 on RSV-triggered NET formation. Treatment of human neutrophils with chloroamidine abrogated the classical NETosis promoted by RSV. The requirement of PAD-4 for NET generation during RSV infection indicates that histone citrullination and chromatin decondensation must occur in order to neutrophils extrude NETs. Intriguingly, the infection of mice with Influenza A virus induces PAD-4-mediated NET formation in the inflamed lung, but these DNA lattices are not required for protection against the virus^14^. In addition to PAD-4, a functional NADPH oxidase activity has been shown to be required to chromatin decondensation that precedes NET release. The inability of neutrophils from CGD patients to produce NETs in response to PMA provides an indirect evidence of the essential role of NADPH oxidase for chromatin decondensation^19^. Nevertheless, the absence of chromatin decondensation when NADPH oxidase is inhibited by the use of DPI directly implies the activity of this enzyme on chromatin decondensation during NETosis^46^. Hence, it is possible that PAD-4 and NADPH oxidase cooperate to promote histone citrullination and chromatin decondensation during RSV-induced NET formation, although a direct relationship between these enzymes remains elusive.

The final step of classical NETosis is the release of NETs via rupture of plasma membrane and cell lysis^47^. Several studies have suggested that NETosis is a type of cell death program different from apoptosis and necrosis^19,29,31,48^. Genetic analysis of neutrophils stimulated with PMA has shown that when NETosis is activated, there is a modulation of antiapoptotic proteins such as Mcl-1, blocking apoptosis^29^. Furthermore, AKT has been reported to switch neutrophil death from apoptosis to NETosis by blocking caspases activation^31^. Taking into consideration that a ruptured plasma membrane is a feature of necrotic cell death and that RSV triggers the activation of a specific intracellular machinery to induce NETosis, we hypothesized that RSV could trigger NET release through the activation of signaling pathways involved in programmed necrosis, also called necroptosis. To test that, neutrophils were treated with specific inhibitors of RIPK1, RIPK3 and MLKL. Indeed, the pretreatment with NEC-1s, a highly selective inhibitor of RIPK-1, completely suppressed NET release induced by RSV. Similarly, pretreating neutrophils with GW42X profoundly decreased NETosis and NSA inhibition of MLKL abolished NETosis promoted by the virus. Therefore, RSV activates the typical necroptosis signaling pathways preceding the release of NETs. Since classical NETosis occurs via cell lysis, we tested whether RSV would be able to induce LDH release as marker of plasma membrane rupture. In fact, RSV infection caused LDH release from neutrophils in a concentration-dependent fashion, confirming that this respiratory virus incites neutrophil necrosis. Interestingly, RIPK1 was not necessary to LDH release promoted by RSV, while the necroptosis executioner pseudokinase MLKL mediated RSV-induced LDH release from neutrophils. MLKL has been recognized as a key functional mediator of necroptosis^49^. Once phosphorylated by RIPK3, MLKL translocates to the plasma membrane and forms membrane-disrupting pores, leading to cell lysis^50^. The implication of necroptosis pathways during NETosis has been a matter of debate. It has been recently demonstrated that NET release occurs when gout-related monosodium urate crystals trigger human and mouse neutrophils to undergo RIPK1-RIPK3-MLKL-mediated necroptosis^25^. Additionally, different microparticles have been reported to induce RIPK1-RIPK3-MLKL-dependent neutrophil necroptosis and that this mode of regulated cell death is associated with NETosis^26^. Furthermore, vasculitis-associated antineutrophil cytoplasmic antibody (ANCA) triggers NET release through the activation of necroptotic pathways, causing endothelial cell damage *in vitro*^51^. On the other hand, GM-CSF in association with LPS or C5A has been shown to stimulate NET formation independently of RIPK3 and MLKL signaling^24^. Intriguingly, the signaling by RIPK3-MLKL has been shown to be both necessary and unnecessary to PMA-induced NET release by these two different groups^24,25^. It is likely that distinct experimental conditions account for those discrepancies in the results. In our experimental settings, we used different inhibitors at the typical concentrations needed to block necroptosis and our data clearly indicates that RSV activates the necroptotic machinery (RIPK1-RIPK3-MLKL) to induce neutrophil lysis that precedes NET release.

Alveolar epithelial cells are the main targets of RSV infection, as well as the first site for the activation of an innate immune response^10^. In order to mimic a respiratory infection, we took advantage of a co-culture model, on which RSV-infected alveolar epithelial cells were exposed to naïve neutrophils. Our aim was to evaluate the effect of RSV infection of alveolar epithelial cells on NET release by human neutrophils. Indeed, neutrophils were able to recognize epithelial cells infected with RSV and to release NETs as a response to the epithelial infection. It is worth noting that free virus particles were removed from the culture system and neutrophils were only exposed to infected epithelial cells. Interestingly, an active RSV infection of alveolar epithelial cells is essential to NET generation, since stimulation of epithelial cells with UV-inactivated RSV was not able to trigger NET-DNA release. Thus, neutrophils identify alveolar epithelial cells actively infected with RSV and respond to infection by releasing NETs. We are currently investigating the mechanisms of neutrophil recognition of infected epithelial cells. To rule out a possible effect of tumor cells (A549 cells) on NET induction during RSV infection, we infected a normal lung fibroblast line and measured NET-DNA release. Our data show that RSV infection of lung fibroblasts was capable of inducing NETosis. NETs contain several antimicrobial molecules capable of damaging endothelial and epithelial cells, as is the case of histones^52^. Furthermore, NETs were also linked with potential lung damage during influenza and rhinovirus infection^15,53^. In fact, patients with severe influenza A infection presented high levels of plasma NET-DNA, which were correlated with the severity of disease and a poor prognosis^54^.

**Figure 7:**
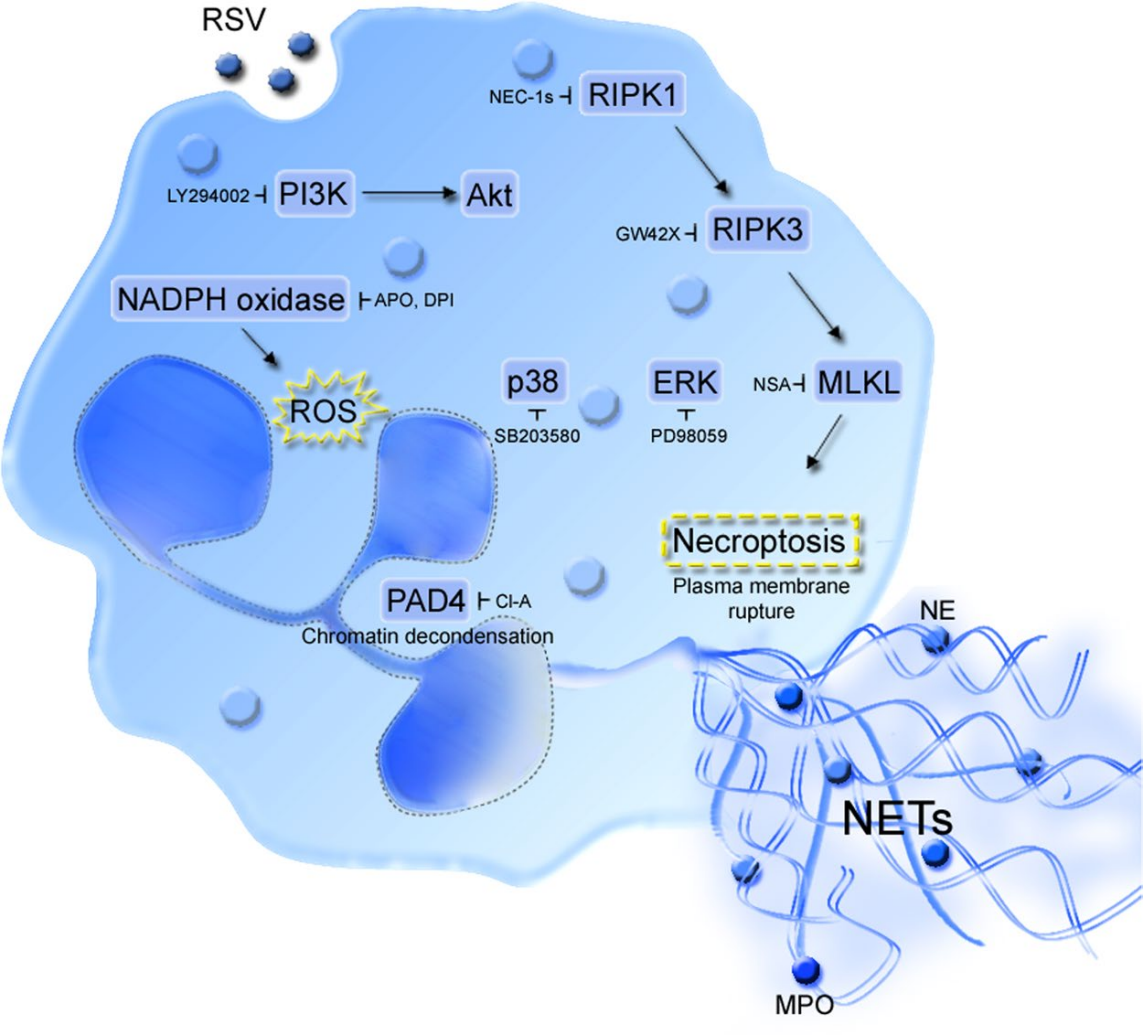
Overview of RSV-triggered classical ROS-dependent NETosis in human neutrophils. RSV infection of human neutrophils leads to ROS generation via NADPH oxidase, which is necessary to NET formation. Additional signaling by PI3K/AKT, ERK and p38 MAPK is required. RSV-induced NETosis requires chromatin decondensation mediated by the enzyme PAD-4. Interestingly, RIPK1-RIPK3-MLKL are key proteins involved in necroptosis, but also essential to RSV-induced NETosis. The activation of these signaling pathways by RSV leads to plasma membrane rupture with the consequent release of decondensed chromatin fibers decorated with NE and MPO, characterizing NETs.

In conclusion, our study demonstrates that RSV infection triggers the classical ROS-dependent NETosis in human neutrophils. This induction needs additional signaling by PI3K/AKT, MAPK and requires histone citrullination by PAD-4. Importantly, we uncover that RSV-induced classical NETosis relies on the activation of the necroptotic machinery, namely RIPK1-RIPK3-MLKL proteins (Fig. 7). Furthermore, neutrophils are able to identify RSV-infected alveolar epithelial cells and lung fibroblasts and to respond by releasing the cytotoxic DNA threads, or NETs. The identification of the signaling cascades responsible to mediate NETosis induced by RSV infection may prove valuable to the design of new therapeutic approaches to treat the devastating inflammatory consequences of RSV bronchiolitis in young children.

## Methods

### Reagents

Diphenyleneiodonium (DPI), apocynin (APO), phorbol 12-myristate 13-acetate (PMA), LY294002, carboxymethylcellulose sodium salt and dextran from Leuconostoc spp. were purchased from Sigma-Aldrich. Hoechst 33342, rabbit anti-Mouse IgG secondary antibody, HRP, goat anti-Mouse IgG1 secondary antibody, PE, Qubit dsDNA HS assay kit and 3-(4,5-Dimethylthiazol-2-yl)−2,5-Diphenyltetrazolium Bromide (MTT) were from Invitrogen. PD98059, SB203580 and Cl-Amidine were from Cayman Chemical. Ham’s F-12 nutrient mix, Opti-MEM, DMEM, RPMI 1640 and fetal bovine serum (FBS) were from Gibco. Anti-neutrophil elastase antibody was from Abcam. Anti-RSV fusion protein antibody and necrosulfonamide (NSA) were from Millipore. Goat anti-rabbit IgG secondary antibody, Cy3 was from Chemicon International. Mouse anti-human myeloperoxidase, PE (MPO) was from BD Biosciences. The 7-Cl-O-Nec-1 (Nec-1s) and GW42X were a gift from Dr. Ricardo Weinlich (Hospital Israelita Albert Einstein, São Paulo, Brazil). Ficoll-Paque PLUS was from GE Healthcare. CytoTox 96 Non-Radioactive Cytotoxicity Assay was from Promega. Falcon 8-well culture slides were from Corning.

### Virus Culture

The virus production of RSV A2 strain (kindly donated by Dr. Fernando Polack, Vanderbilt University School of Medicine, USA) was obtained in VERO cells cultured in Opti-MEM medium with 2% FBS at 37 °C under 5% CO_2_. To assess viral titer, VERO cells were infected with RSV in medium without serum followed by a carboxymethylcellulose plaque assay. To assess the viral titer on supernatants of RSV-infected, uninfected or UV-RSV-treated A549 cells, VERO cells were incubated with these supernatants without serum, followed by a carboxymethylcellulose plaque assay. Lysis plate titration was performed using an anti-RSV antibody and viral titer was expressed as plaque forming units (PFU). The virus aliquots were stored at −80 °C.

### Bacterial culture

*Staphylococcus aureus* ATCC 29213 was grown in Brain Heart Infusion (BHI) broth (Oxoid) for 20 h at 37 °C until stationary phase. To determine the number of colony-forming units per milliliter (CFU/mL) of the culture, a 1 mL-aliquot was centrifuged at 10,000 × *g* for 4 min and the pellet resuspended in 1 mL of 0.85% sterile saline. Afterwards, bacterial pellet was serially diluted until 10^−8^ and spotted as 10 μL of each dilution on nutrient agar (Oxoid) in triplicate, and incubated at 37 °C for 24 h. Bacteria were added to neutrophils in RPMI 1640 medium at bacteria to neutrophil ratios of 10:1 (determined from Pilsczek *et al*.^21^) and incubated for 10 minutes at 37 °C under 5% CO_2_.

### Human Neutrophil Isolation

Whole blood (20 mL) was collected from healthy volunteer donors (with a mean age of 28 years, from both sexes) into heparin-treated tubes. Neutrophils were purified by density gradient centrifugation using Ficoll-Paque PLUS. Erythrocytes were removed by dextran sedimentation followed by two rounds of hypotonic lysis. Purified neutrophils were re-suspended at 2 × 10^6^ cells/mL in RPMI 1640 medium. Neutrophil viability was assessed by the trypan blue exclusion assay and was always higher than 98%.

### Stimulation of neutrophils and quantification of NETs

Neutrophils (2 × 10^6^ cells/mL) were stimulated with PMA (50 nM), RSV A2 strain (10^2^–106 PFU/mL) or UV-inactivated RSV (10^4^ PFU/mL) for either 10 or 180 minutes at 37 °C under 5% CO_2_. As a control for rapid extracellular DNA release, neutrophils were stimulated with *S. aureus* at bacteria to neutrophil ratios of 10:1 for 10 minutes at 37 °C with 5% CO_2_. After the stimulation period, culture supernatant was collected and extracellular DNA was measured using the dsDNA Picogreen kit or Quant-iT dsDNA HS kit (both from Invitrogen), following manufacturer’s instructions and obtaining similar results. Therefore, the DNA measured is referred to as NETs. To evaluate the role of specific signaling pathways on RSV-induced NET release, neutrophils were pretreated for 1 hour with selective inhibitors: apocynin (APO; 10 µM), diphenyleneiodonium (DPI; 10 µM), LY294002 (LY; 50 µM), PD98059 (PD; 30 µM), SB203580 (SB; 10 µM), Cl-Amidine (Cl-A; 12 µM), necrostatin-1s (Nec-1s; 50 µM), GW42X (GW; 0.5 µM) or necrosulfonamide (NSA; 5 µM). The Trypan Blue exclusion assay was used to evaluate the viability of cells treated with these inhibitors and at the end of incubation, cell viability was always higher than 97%. Due to the variability in the human donors’ response, all data were presented as n fold negative control.

### Immunofluorescence

Neutrophils (1 × 10^5^/300 μL) were seeded in 8-chamber culture slides and incubated with RSV for 180 minutes at 37 °C under 5% CO_2_. Afterwards, cells fixed with 4% paraformaldehyde (PFA) were stained with anti-RSV fusion protein (1:1000) followed by anti-mouse PE antibody (1:500), anti-elastase (NE; 1:1000) followed by anti-rabbit Cy3 antibodies (1:500) or anti-myeloperoxidase PE antibody (MPO; 1:1000) and Hoechst 33342 (1:2000). Images were taken in a confocal Zeiss LSM 5 Exciter microscope.

### Assay of intracellular ROS generation

The determination of intracellular ROS generation was based on the oxidation of 0.5 µM 5-(and-6)-chloromethyl-2′, 7′-dichlorodihydrofluorescein diacetate, acetyl ester (CM-H_2_DCFDA) to yield an intracellular fluorescent compound. Neutrophils (2 × 10^6^ cells/microtube) were pretreated with DPI (10 µM) and stimulated with PMA (50 nM) or RSV (10^4^ PFU /mL) for 60 minutes at 37 °C under 5% CO_2_. Afterwards, cells were incubated with CM-H_2_DCFDA for 30 minutes at 37 °C with 5% CO_2_. Cytosolic ROS production was measured by flow cytometry, using FACSCanto II flow cytometer (Becton Dickinson) with the BD FACSDiva software and analyzed with FlowJo v 7.5.

### A549 and MRC-5 cell culture and co-culture with neutrophils

Lung adenocarcinoma epithelial cell line (A549 cell) and non-tumor human lung fibroblast cell line (MRC-5 cell) were grown in monolayers and maintained in Ham’s F-12 nutrient mix or DMEM medium, respectively, supplemented with 10% FBS at 37 °C in 5% CO_2_. A549 or MRC-5 cells (1.5 × 10^5^ cells/ mL) were infected with different concentrations of active or UV-inactivated RSV (10^2^–104 PFU /mL) in RPMI medium for 48 hours at 37 °C in 5% CO_2_. After this period, the medium was replaced to remove any free virus particles, human neutrophils were added to the cultures at the ratio of 1 epithelial cell/fibroblast: 5 neutrophils and the co-culture was maintained for 180 minutes at 37 °C under 5% CO_2_. Afterwards, the culture supernatants were collected and extracellular DNA was measured using the Quant-iT dsDNA HS kit, following manufacturer’s instructions.

### Cytotoxicity Measurements

Cellular cytotoxicity was assessed by detecting LDH in neutrophil supernatants stimulated with RSV (10^2^–106 PFU/mL) for 180 minutes using the CytoTox 96 Non-Radioactive Cytotoxicity Assay kit according to manufacturer’s instructions. Readings were carried out at 490 nm wavelength, using EZ Read 400 microplate reader (Biochrom).

### Cell Viability Assay

MTT assay was performed in A549 cells infected with different concentrations of RSV (10^2^–10^4^ PFU/mL) for 24, 48 and 72 hours. Briefly, MTT solution (40 µL; 5 mg/mL) was added to the culture, which was maintained for 4 hours at 37 °C in 5% CO_2_. After this period, MTT solution was removed and DMSO (120 µL) was added to cells. Cell viability was assessed using EZ Read 400 microplate reader (Biochrom) at 570 nm wavelength.

### Statistical Analyses

Data were presented as mean ± SEM of values for 3 independent replicates of independent experiments. The results obtained were analyzed using GraphPad Prism 6 statistical software package. Comparisons between multiple groups were analyzed with one-way ANOVA and a posteriori Bonferroni test. When appropriate, unpaired Student’s t-test or Mann Whitney test were employed. The level of significance was set at p ≤ 0.05.

### Ethics Statement

This study was carried out in accordance with the recommendations of the Research Ethics Committee of Pontifical Catholic University of Rio Grande do Sul (CEP/PUCRS). The protocol was approved by the Research Ethics Committee of Pontifical Catholic University of Rio Grande do Sul (CEP/PUCRS) under protocol number CEP 1.743.173. All subjects gave written informed consent in accordance with the Declaration of Helsinki.
